# Household expenditure of smokers and ex-smokers across socioeconomic groups: results from a large nationwide Australian longitudinal survey

**DOI:** 10.1186/s12889-022-14083-y

**Published:** 2022-09-08

**Authors:** Anita Lal, Mohammadreza Mohebi, Sarah L. White, Michelle Scollo, Nikki McCaffrey

**Affiliations:** 1 Deakin Health Economics, Institute for Health Transformation, Deakin University, Geelong, Australia; 2 Biostatistics Unit, Faculty of Health, Deakin University, Geelong, Australia; 3 Quit, Cancer Council Victoria, Melbourne, Australia; 4 Cancer Council Victoria, Melbourne, Australia

**Keywords:** Smokers, Quitters, Smoking cessation, Household expenditure

## Abstract

**Background:**

Countries with best practice tobacco control measures have experienced significant reductions in smoking prevalence, but socioeconomic inequalities remain. Spending on tobacco products, particularly by low-income groups can negatively affect expenditure on other goods and services. This study aims to compare the household expenditure of adults who smoke tobacco products and those who formerly smoked across socioeconomic groups.

**Methods:**

Daily smokers and ex-smokers were compared using the Household, Income and Labour Dynamics in Australia Survey, over 7 waves. Adults who never smoked were not included. Participants were continuing sample members across waves. Mean number of participants per wave was 2505, 25% were smokers and 75% ex-smokers. The expenditure variables investigated included tobacco products, alcohol, motor vehicle fuel, health practitioners, insurance, education, and meals eaten out. Regression models using the generalized estimating equation technique were employed to compare expenditure data aggregated across the waves by Socioeconomic Index for Areas (SEIFA) quintiles of relative socio-economic advantage/disadvantage while accounting for within-participant autocorrelation. Quintiles are ranked by information such as the income, occupation and access to material and social resources of the residents.

**Results:**

Smokers from all quintiles spent significantly less per year on meals out, education and insurance than ex-smokers (*p* < 0.001). Smokers from quintiles 2–5 spent less on groceries, medicines, and health practitioners (*p* < 0.01). Smokers from quintiles 1 and 2 (most disadvantaged), spent less on motor vehicle fuel than ex-smokers ($280;95%CI: $126–$434**),** ($213;95%CI: $82–$344). Smokers from quintiles 2 and 3 spent more on alcohol ($212;95%CI: $86–$339), ($231.8;95%CI: $94–$370) than ex-smokers. Smokers from the least disadvantaged groups spent less on clothing than ex-smokers ($348;95%CI: $476–$221), ($501; 95%CI: $743–$258). Across the whole sample, smokers spent more than ex-smokers on alcohol ($230;95%CI:$95–$365) and less on meals out ($361;95%CI:$216–$379), groceries ($529;95%CI:$277–$781), education ($456;95%CI:$288–$624), medicine ($71;95%CI:$38–$104), health practitioners ($345;95%CI:$245–$444) and insurance ($318;95%CI:$229–$407).

**Conclusions:**

Smoking cessation leads to reallocation of spending across all socioeconomic groups, which could have positive impacts on households and their local communities. Less spending on alcohol by ex-smokers across the whole sample could indicate a joint health improvement associated with smoking cessation.

**Supplementary Information:**

The online version contains supplementary material available at 10.1186/s12889-022-14083-y.

## Introduction

Countries that have adopted best practice tobacco control measures have experienced significant reductions in smoking rates [1]. However, inequalities in smoking prevalence across socioeconomic position (SEP) remain [2–4]. In 2019 the percentage of daily smokers in Australia was 34% in the lowest socioeconomic group, compared to 9% in the highest [5]. There are also disparities in the number of cigarettes smoked daily by SEP, with the most disadvantaged group in Australia smoking on average about 40 more cigarettes per week [6]. Spending on tobacco products, particularly by disadvantaged individuals and households, can negatively affect expenditure on other goods and services.

Several international studies report on the likelihood of financial stress and smoking-induced deprivation (spending money on cigarettes instead of on household essentials) among smokers compared with non-smokers [7–9]. Financial stress is measured using survey items such as being unable to pay utility bills on time or going without meals. In Australia, the probability of experiencing smoking-induced deprivation is greater for those from low-income groups [7, 10]. Late bill payments, going without meals and having insufficient money for petrol, clothing and family leisure activities are some of the ways smoking displaces other spending [11]. Experiencing financial stress is more prevalent among smokers than ex-smokers or never smokers [12, 13].

Smoking cessation may lead to positive changes in household spending; however, few studies have compared the spending of smokers and ex-smokers. The most current research from Australia is based on data from 2006 showing that ex-smokers from low SEP groups, who had made changes to spending, spent more on food and clothing [14]. Ex-smokers in 2001–2005 had a 25–42% reduction in the odds of financial stress [15]. More recently in the US, when ex-smokers were tracked over a one-year period, the lowest SEP groups had significantly lower spending on alcohol, food at home, transportation and entertainment [16]. Compared to Australia, the US has at least 30% lower excise tax rates on cigarettes as well as less smoke-free restrictions in hospitality venues.

Changes in the discretionary income of adults who quit may be just one factor influencing purchases. Tobacco use has been strongly linked with other health-related behaviours, including higher alcohol consumption and less expenditure on food. Smoking cessation is associated with changed eating patterns and increases in food intake [17]. There are neurobiological mechanisms that make co-administration of nicotine and alcohol intake more pleasurable [18]. In addition, smokers can consume more alcohol due to nicotine being able to directly offset its sedative properties. Smoking also counteracts the cognitive deficits associated with alcohol intoxication in the short term [19]. Alcohol consumption also has the effect of reducing the usual brakes on smoking whereby people try to minimise their consumption. This may therefore result in ex-smokers reducing or avoiding alcohol [20, 21].

To our knowledge, the expenditure patterns of smokers compared to ex-smokers by SEP group in Australia has not been previously investigated using quantitative data. We explored several household expenses such as groceries, utilities, alcohol, meals eaten out and healthcare. Our study aimed to compare the household expenditure of smokers and ex-smokers across socioeconomic groups in Australia using data over an 7-year period. This analysis will provide insights into whether smoking cessation may help to reallocate household expenditure in a way that benefits economic and health outcomes.

## Methods

### Study sample

We used seven waves of the Household, Income and Labour Dynamics in Australia (HILDA) Survey; a nationally representative longitudinal study based on household samples living in private dwellings [22]. Data collection began in 2001 and the sample has been extended to include new members of the original household as well as sample replenishment with additional households added in wave 11. Data are collected by interviewer questionnaire as well as self-completion questionnaires. We obtained our sample from participants responding in the years 2012 to 2018 (waves 12–18). Included in our sample were adult smokers and ex-smokers and being continuing sample members across waves. Data from a total of 17,605 adults were included in this analysis.

### Variables

#### Primary independent variable

We used the subsamples of smokers and ex-smokers. Respondents who indicated they smoked tobacco daily at each wave were categorised as smokers. Those who indicated they no longer smoked were classified as ex-smokers. Participants may have changed category if their smoking status changed.

#### Dependent variables

We analysed household expenditure variables that were collected in waves 12–18. The dependent variables included yearly expenditure on cigarettes and other tobacco products, groceries, alcohol, transport, meals eaten out, motor vehicle fuel, clothing, utilities such as telephone, electricity and gas, internet, education fees (for themselves or their dependents), medicines, health practitioners, and insurance (home, contents and motor vehicle). Expenditure was captured as a continuous variable. The imputation process was carried out by the researchers conducting the survey [23], the details can be found in Additional file 1. Expenditure was adjusted to 2018 dollars using the Australian Institute of Health and Welfare Health price inflators for healthcare related expenditure [24], and the Consumer Price index from the Australian Bureau of Statistics [25].

#### Socio-demographics and potential confounders

We considered several potential confounding variables which are commonly reported in smoking and financial stress studies. This included age, gender, having children, marital status, and remoteness scale (major city, inner regional, outer regional, remote and very remote). Respondents were categorised according to the Australian Bureau of Statistics (ABS) Socioeconomic Index for Areas (SEIFA), and Index of relative advantage/ disadvantage and Index of Education and Occupation (IEO) [26]. The SEIFA quintiles represent groups of individuals who live in similarly ranked areas, based on a range of information such as the income, qualifications, and occupation skills of the area residents [26]. Socio-economic advantage and disadvantage is defined by the ABS in terms of people’s access to material and social resources, and their ability to participate in society [27]. The Index of Education and Occupation (IEO) reflects the educational and occupational level of communities by level of qualification and skill levels of the Australian and New Zealand Standard Classification of Occupations and the unemployed [28].

#### Analyses

Overall mean expenditure and the chosen expenditure categories across waves 12–18 among smokers and ex-smokers were inspected visually using box plots for the presence of extreme and out-of-range values. Summary measures including median and interquartile range (IQR) were used to describe expenditure categories. Regression models using the generalized estimating equation (GEE) technique were employed to aggregate data across the survey waves 12–18 to account for within-participants autocorrelation, to address the longitudinal nature of the study. Separate models for SEIFA and IEO quintiles were employed [29]. A robust sandwich estimator was used for model variance estimation and an unstructured covariance structure was employed to account for within-participant autocorrelation. Effects of age, gender as time-invariant and having children, marital status, and remoteness scale as time-updating (i.e., updating across the waves) variables were initially investigated through GEE models and excluded from the final models due to non-significant *p*-values. Two-way interactions between smoking status and confounding variables were investigated in additional GEE models. The models with significant interactions were depicted graphically. The SEIFA quintiles of relative socio-economic advantage/disadvantage and IEO were investigated in separate GEE models to avoid autocorrelation. Two tailed *p*-values ≤0.05 were considered significant for overall across SEIFA /IEO quintiles comparisons, and the Bonferroni approach was used to account for type I error inflation due to multiple comparisons for all within quintiles comparisons (*p* ≤ 0.01). Stata 16 was used for data analysis [30].

## Results

### Descriptive Statistics

The percentage of smokers ranged in each wave from 28% (2012) to 23% (2018) and 72% to 77% ex-smokers. The total numbers vary slightly in each wave due to new entrants being added or leaving a continuing sample member household, death or moving overseas (Table 1).

**Table 1:** Number of participants in each wave 2012–2018

Year	Daily smokers	Ex-smokers	Total study sample	Percentage of HILDA sample
2012	694 (28%)	1813 (72%)	2507	26%
2013	666 (26%)	1863 (74%)	2529	26%
2014	656 (26%)	1863 (74%)	2519	26%
2015	609 (24%)	1902 (76%)	2511	26%
2016	610 (24%)	1925 (76%)	2535	27%
2017	581 (23%)	1919 (77%)	2500	26%
2018	568 (23%)	1936 (77%)	2504	26%

Numbers may not add to 100% due to rounding. Categories are reported as described and were combined for modelling.

Table 2 presents the demographics of participants in wave 12. Around half the participants were female for both smokers (51.0%) and ex-smokers (49.4%). Ex-smokers tended to be older and part of a couple without children. A greater percentage of the smokers were lone persons (27.9% versus 20.1%) and single parents (9.1% versus 3.9%). More ex-smokers had a university degree (23.5% versus 9.7%). The median income level was around $10,000 higher in the ex-smokers’ group.

**Table 2:** Characteristics of the subsample of smokers and ex-smokers at first wave of analysis (wave 12), n-2507

Characteristic	Smokers % (*n* = 694)	Ex-smokers % (*n* = 1813)	all % (*n* = 2507)
**Gender**			
Women	51.0	49.4	50.1
Men	49.0	50.6	49.9
**Age**			
25–39	26.1	11.7	15.7
40–54	44.7	33.9	36.9
≥ 55	29.2	54.4	47.4
**Household type**			
Couple without children	32.4	47.2	43.1
Couple with children	26.7	26.4	26.5
Lone person	27.9	20.1	22.3
Single parent	9.1	3.9	5.4
Single parent living with others	0.3	0.2	0.2
Other family no children	1.0	0.6	0.7
Group household	0.7	0.7	0.7
Multi-family household	1.9	0.9	1.2
**Education**			
Did not complete high school	37.9	30.7	32.7
Completed High school	13.0	10.2	11.0
Diploma or Certificate	39.5	35.6	36.7
University or other higher education	9.7	23.5	19.7
**Income**			
Median (interquartile range)	$76,065 ($44,000–$116,319)	$86,700 ($42,370–$142,484)	$83,556 ($42,862–$134,166)
< 25,000	11.5	9.9	10.3
25,000–49,000	17.6	20.6	19.7
50,000–74,999	20.3	13.3	15.2
≥ 75,000	50.6	56.3	54.7

### Household expenditure

Across all seven waves expenditure for smokers was significantly higher than ex-smokers for cigarettes and tobacco (+$5045) and alcohol (+$230), and significantly lower in the expenditure categories of eating out (−$361), groceries (−$529), education (−$456) medicines (−$108), health practitioners (−$345) and general insurance (−$318) (*p* < 0.001) (Table 3). For the categories of utilities, clothing, internet and phone, public transport and motor vehicle fuel, overall mean expenditure was not significantly different amongst smokers and ex-smokers. Additional file 2 details the median expenditure by quintile per annum across waves.

**Table 3:** Mean expenditure differences of smokers and ex-smokers by socioeconomic position per annum

	Alcohol	Meals eaten out	Groceries	Education	Clothing	Motor Vehicle Fuel	Medicine	Health Practitioner	Insurance
Total sample	$230.0* **($95.3–$364.7)**	−$361.2** **(− $378.9−− $215.9)**	− $528.8** **(− $780.6−− $277.1)**	−$456.0** **(− $624.0−− $288.1)**	−$181.6 (− $386.9–$23.8)	−$152.5 (− $297.2−−$7.8)	−$70.9* **− $103.6−−$38.3**	−$344.7** **(−$444.5−−$245.0)**	−$318.1** **(−$407.4−− $228.9)**
SEIFA quintile 1	$192.8 ($27.0–$358.7)	−$355.5** **(− $512.6−− $198.5)**	− $329.4 (− $648.3−− $10.4)	− $276.9** **(− $412.4−− $141.3)**	$109.06 (−$ 409.2−− $627.3)	**−**$279.9** **(− $434.2−− $125.6)**	−$50.1 (−$93.4 −− $6.8)	−$132.4 (− $248.0−− $16.7)	− $291.0** **(− $397.6−− $184.4)**
SEIFA quintile 2	$212.3* **($86.0–$338.6)**	−$358.5** **(−$476.3−− $240.7)**	−$434.0** **(− $672.5−− $195.5)**	−$370.8** **(− $494.3-−− $247.3)**	−$43.3 (−$ 391.4–$304.7)	**−**$213.1* **(− $344.3−− $81.8)**	−$61.0** **(− $92.8−− $29.3)**	−$243.7** **(− $334.6−− $152.8)**	− $305.2** **(− $386.5−− $224.0)**
SEIFA quintile 3	$231.8* **($93.8–$369.8)**	−$361.5** **(− $493.3−− $229.6)**	− $538.6** **(− $796.6−− $280.5)**	− $464.8** **(−$ 639.0-−− $290.5)**	−$195.8 (− $388.2−−$ 3.4)	−$146.2 (− $294.1−− $1.5)	− $72.0** **(− $105.4 −− $38.5)**	−$355.1** **(− $457.3−− $252.8)**	−$319.5** **(− $411.1−− $227.8)**
SEIFA quintile 4	$251.3 ($59.3–$443.2)	−$364.5** **(− $551.9−− $177.0)**	−$643.1** **(− 1004.8−− 281.5)**	− $558.7** **(− $811.2−− $306.3)**	−$348.2** **(− $475.6−− $220.7)**	−$79.5 ($273.5−− $114.6)	−$ 82.9** **(− $129.9−−$ 35.9)**	−$466.5** **(− $607.8−− $325.2)**	− $333.7** **(−$ 463.2−− $204.2)**
SEIFA quintile 5	$270.7 ($7.47–$534.0)	−$367.4** **(− $626.6−− $108.2)**	− $747.7* **(− $1247.2−− $248.3)**	− $652.7** **(− $992.2−− $313.1)**	−$500.6** **(− $743.1−−$ 258.1)**	−$12.6 (− $266.9−− $241.6)	−$93.8** **(− $159.2−− $28.5**	−$-577.8** **(− $769.6−− $386.1)**	− $347.9** **(− $526.0−− $169.8)**
IEO quintile 1	$191.9 ($80.5–$341.6)	−$383.6** **(−$556.6−− $210.5)**	−$347.1 (− $664.2−− $30.0)	− $284.8** **(− $447.2−−$ 122.4)**	−$122.7 (− $345.0–$99.7)	−$221.7* **(− $387.9−− $55.5)**	−$64.0** **(− $109.3−− $18.7)**	−$252.1** **(− $386.4−− $117.9)**	−$276.3** **(− $381.3−− $171.3)**
IEO quintile 2	$201.6* **($73.8–$329.4)**	−$394.0** **(− $523.5−− $264.6)**	− $452.5** **(− $694.5−− $210.4)**	− $372.2** **(− $508.5−− $1236.0)**	$302.4 (− $644.7–$1249.5)	−$194.6 (− $410.3−− $21.1)	−$79.7** **(− $113.2−− $46.1)**	− $309.5** **(− $409.7−− $209.3)**	−$305.1** **(− $388.4−− $221.7)**
IEO quintile 3	$211.2* **($80.2–$342.2)**	−$404.5** **(−$526.4−− $282.6)**	− $557.9** **(−$ 805.0−− $310.7)**	− $459.6** **(− $616.5−− $302.8)**	−$280.1 (− $494.4−− $65.7)	−$50.2 (− $327.9–$227.5)	− $95.4** **(− $126.9−− $63.8)**	−$366.9** **(− $463.5−− $270.3)**	−$333.8** **(− $419.9−− $247.7)**
IEO quintile 4	$220.8 ($52.4–$389.3)	−$414.0** **(− $570.7−− $259.3)**	− $663.3** **(− $991.9−− $334.6)**	− $547.0** **(− $757.9−− $336.2)**	−$457.7** **(− $683.0−− $232.3)**	$136.9 (− $285.2–$559.1)	− $111.0** **(− $151.7−− $70.4)**	−$424.2** **(− $550.2−− $298.3)**	− $362.5** **(− $474.1−− $251.0)**
IEO quintile 5	$230.5 ($7.0–$454.0)	−$425.5** **(− $637.4−− $213.6)**	− $768.7** **(− $1215.2−−$322.1)**	− $634.5** **(−$914.1−−$354.9)**	−$310.6* **(− $548.5−− $72.8)**	−$229.1 (− $537.1–$78.9)	− $126.7** **(− $182.4−− $71.0)**	−$481.6** **(− $653.8−− $309.4)**	−$391.3** **(− $539.7−− $242.9)**

Mean expenditure difference is the mean of the difference in expenditure between smokers andex-smokers. Bold font indicates significance

*n* = 17,605. Covariates included age, gender, having children, marital status, and remoteness scale

*SEIFA* Socioeconomic Index for Areas, *IEO* index of education and occupation

^*^p < 0.01

^**^p < 0.001

### Within quintile results

**Fig. 1:**
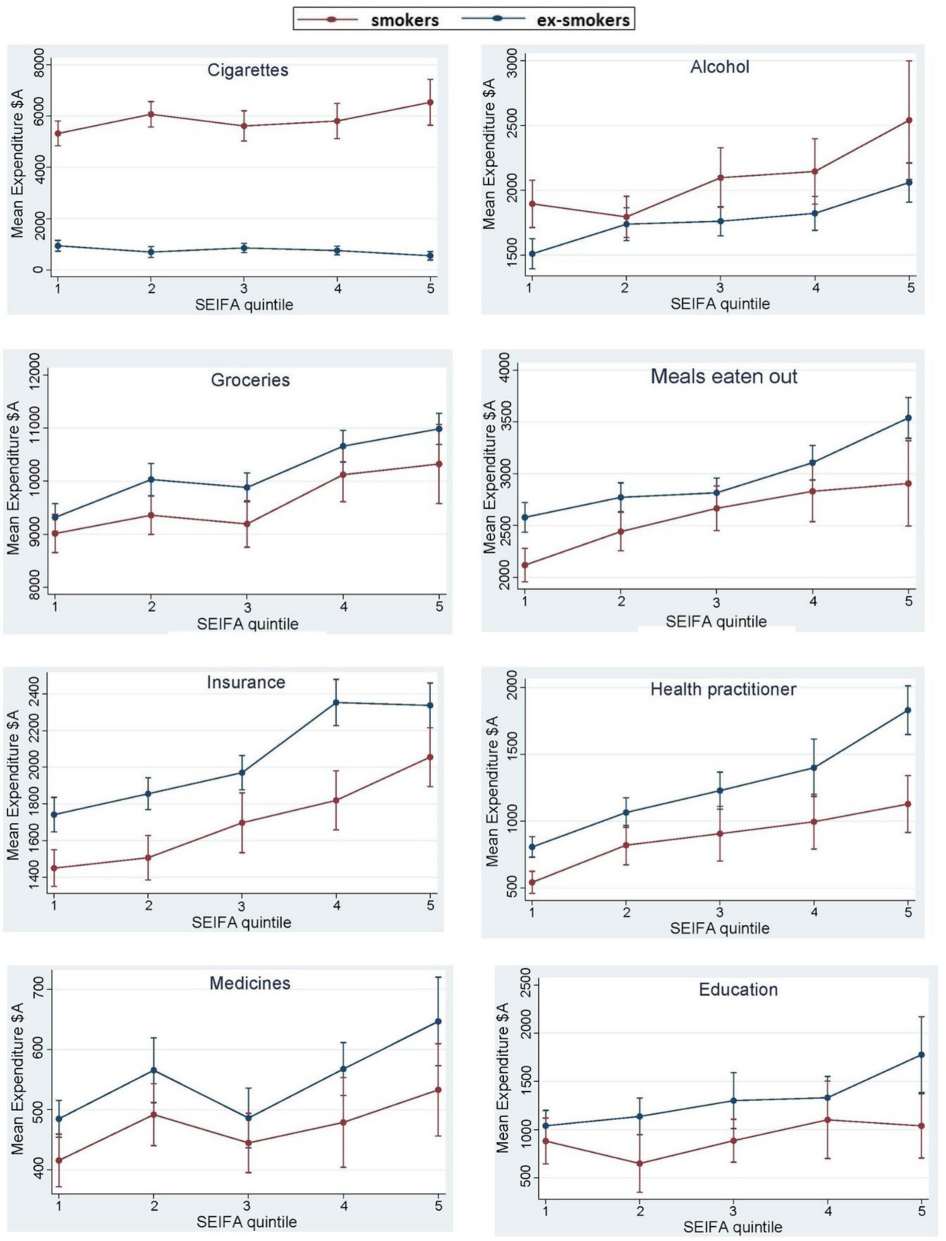
Smokers and ex-smokers mean expenditure per annum ($A) and 95% CI by Socioeconomic Index for Areas (SEIFA) quintile, averaged across waves. Notes: line indicates smokers; the blue line indicatesa ex-smokers, the lower SEIFA quintile represents more disadvantage

**Fig. 2:**
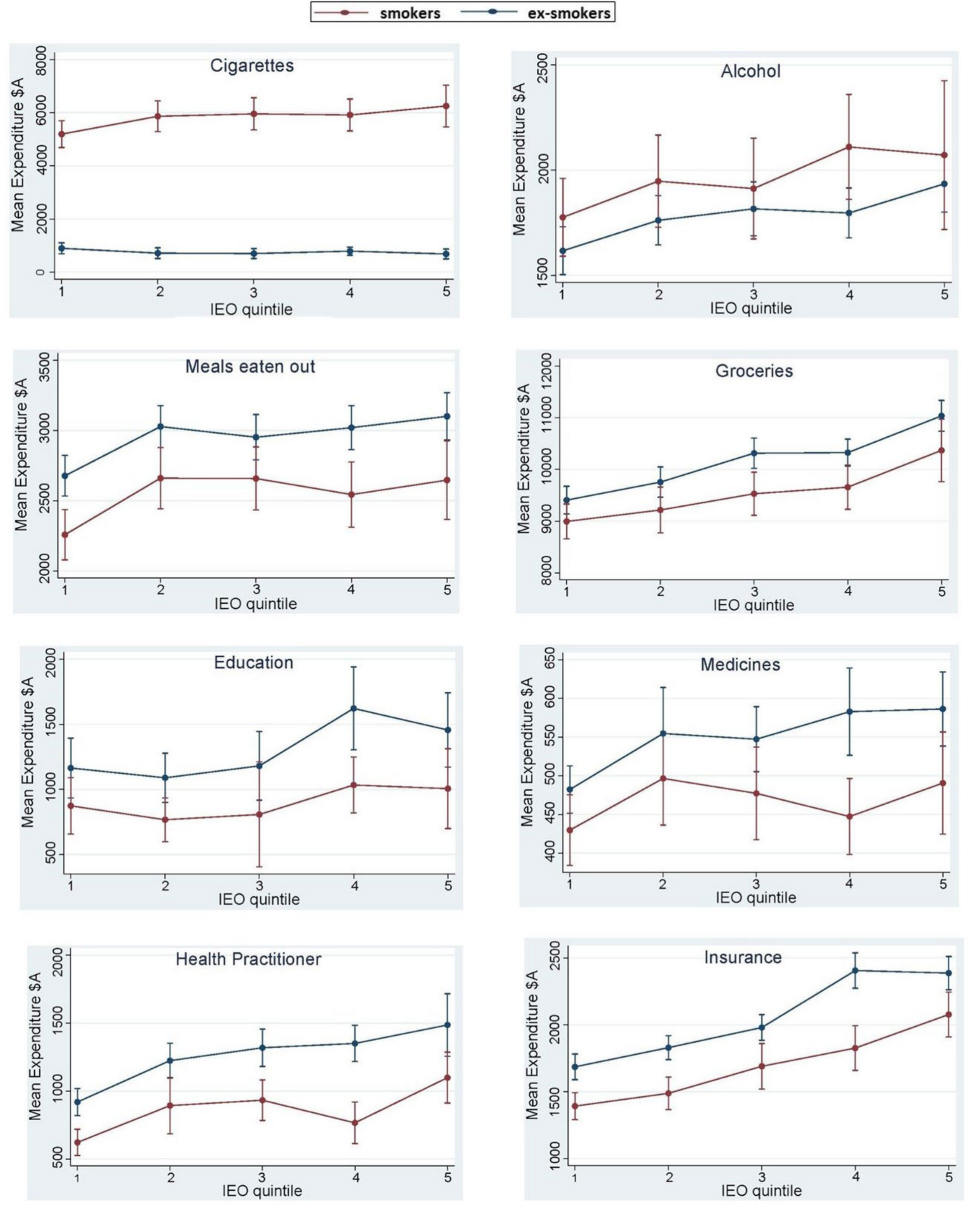
Smokers and ex-smokers mean expenditure per annum ($A) and 95% CI by Index of Education and Occupation (IOE) quintile, averaged across waves. Notes: Red line indicates smokers; the blue line indicates ex-smokers, the lower IEO quintile represents lower education/occupation

For models with overall significantly different expenditure patterns between smokers and ex-smokers, average expenditure across waves is presented in Figs. 1 and 2. Figures 1 and 2 illustrate marginal mean predictions of expenditure from models that included significant two-way interactions of socioeconomic quintile and smoking status. The blue line, representing the ex-smokers, is mostly above the red line, indicating lower mean expenditure by smokers. For cigarettes and tobacco, and alcohol, the blue line is lower than the red line, indicating lower mean expenditure by ex-smokers. Significant interactions are detailed in the following categories.

#### Motor vehicle fuel

Expenditure on motor vehicle fuel was significantly lower for smokers in quintile 1 and quintile 2 compared to ex-smokers, with mean differences of −$280 and −$213 respectively (*p* < 0.01). There was a significant interaction between smoking status and age. As each year of age increased, smokers spent an additional $10 (95% CI: $0–$20) on fuel than same age ex-smokers.

#### Cigarettes and tobacco

The difference in expenditure on cigarettes and tobacco between smokers and ex-smokers in quintiles 1–5 ranged from +$4334 in quintile 1 (most disadvantaged) to +$5949 in quintile 5 (least disadvantaged) (*p* < 0.01). Quintile 2 had the second highest difference in expenditure on cigarettes and tobacco (+$5328).

#### Alcohol

In quintiles 2 and 3, mean expenditure on alcohol was significantly higher for smokers than ex-smokers by $212 and $231 respectively (*p* < 0.01). In all other quintiles the results were not significant.

#### Meals eaten out

Expenditure on meals eaten out was significantly lower for smokers than ex-smokers in all quintiles with mean differences ranging from −$356 in quintile 1 to −$367 in quintile 5 (*p* < 0.01).

#### Groceries

Apart from quintile 1, expenditure on groceries was lower for smokers than ex-smokers, with mean differences ranging from −$434 in quintile 2 to −$747 in quintile 5 (*p* < 0.001). There were significant interactions between smoking status and remoteness index and smoking status and marital status: smokers living in outer regional areas, spent $720 (95% Confidence Interval (CI): $18. − $1422) more than ex-smokers living in major cities, and married smokers spent $487 (95% CI: −$969−−$5) less than unmarried ex-smokers.

#### Education

Mean difference in expenditure on education was significantly lower for smokers than ex-smokers in all quintiles with differences in the range of −$276 to −$653 (*p* < 0.001). There were significant interactions between smoking status and age, smoking status and gender and smoking status and having children. As each year of age increased, smokers spent an additional $15 (95% CI: $5–$26) on education compared with same age ex-smokers. Female smokers spent $276 less (95% CI:−$548−−$5) on education compared with male ex-smokers. Smokers with children spent $393 (95% CI: −$686−−$100) less on education than ex-smokers without children.

#### Clothing

Expenditure on clothing by smokers was significantly lower for quintiles 4 and 5, than ex-smokers with mean differences of −$348 and − $500 respectively per year (*p* < 0.001).

#### Medicine

Apart from quintile 1, expenditure on medicines was significantly lower for smokers than ex-smokers, with mean differences ranging from $61 to $94 per year (*p* < 0.001).

#### Health practitioner

Apart from quintile 1, smokers had significantly lower expenditure on health practitioners than ex-smokers, with mean differences ranging from −$244 to −$577 (*p* < 0.001). There was a significant interaction between smoking status and remoteness index: adults living in outer regional areas who smoke, spent an additional $393 (95% CI: $185–$600) on health practitioners than adults living in major cities who quit smoking.

#### Insurance

The mean difference in spending on insurance was significantly lower for smokers than ex-smokers in all quintiles ranging from −$291 to −$348 (*p* < 0.001). There were significant interactions between smoking status and remoteness index and smoking status and marital status. Smokers living in remote areas spent $804 less (95% CI: −$1508−−$100) than ex-smokers living in major cities. Married smokers spent $163 (95% CI: −$310−−$16) less than unmarried ex-smokers.

## Discussion

This study aimed to compare household expenditure of smokers and ex-smokers, particularly among the lowest SEP groups. We found that in the lowest SEP group (quintile 1), smokers spent on average $4335 per annum on cigarettes and tobacco products and ex-smokers spent more on meals eaten out, education, motor vehicle fuel and insurance than smokers. Ex-smokers in SEIFA quintile 2 had the most differences in spending categories, with more spending in the same categories as quintile 1, along with groceries, medicine and health practitioners and less on alcohol. The apparent expenditure shifts from tobacco to spending in other areas indicate there are societal benefits of smoking cessation, beyond the direct health effects in the form of increased expenditure in the local community, such as to grocery stores and restaurants and cafes. For the Australian economy, spending on other goods and services is beneficial and would increase the income and prosperity for local businesses given that tobacco companies are transnational. Our results indicate that the interaction effects of marital status, having children, remoteness index and gender on cigarettes, alcohol, meals eaten out and medicine expenditure were not significant.

Less expenditure on alcohol by ex-smokers indicates a possible joint health effect improvement: smoking cessation and reduced alcohol intake. Our results highlight previous research that nicotine increases alcohol reinforcement and cravings in smokers and its absence results in a decrease in consumption [31, 32]. Greater alcohol expenditure in smokers may also reflect previous findings that they are able to consume more due to nicotine directly offsetting the cognitive deficits associated with alcohol intoxication [33]. Alcohol consumption is also related to relapses in smoking cessation attempts [20]. Our findings of a reduction in alcohol expenditure support prior research from a longitudinal study that examined recent adults who quit over a 12-month period from the U.S. [16] Rogers et al also found that quitting reduced households expenditure on other items that facilitate or complement smoking cessation such as food, entertainment, and transport [16]. However, our findings indicate an increase in eating out for adults who quit. Rogers et al considered restaurants to be a smoking trigger; households with a smoker who had relapsed tended to have higher spending on food away from home. Differences in our findings may be due to smoke-free restaurants and cafes being more regulated in Australia. Expenditure on food at home was not collected as part of the HILDA survey so the extent to which overall food expenditure has changed is not known. Our results could indicate that adults who quit switched from consuming alcohol to eating out.

For adults who quit, higher health expenditure on health practitioners and medicines was found overall. Smoking cessation often occurs during a costly healthcare episode which prompts them to quit [34]. This has been found by several previous studies, that followed smokers and ex-smokers over several years [34–37]. Ex-smokers showed an increase in healthcare use and costs that began just before cessation and further increase after cessation, often over a period of 1 year, with rates of healthcare utilisation declining in subsequent years. Within 4-7 years after cessation, adults who quit returned to their baseline levels of healthcare use or lower [34, 35]. Another possible explanation could be that lower socioeconomic quintiles prioritise purchasing tobacco products over medications or that they forego seeing a health practitioner due to the expense. However, for health practitioner expenditure there was a significant interaction between smoking status and remoteness index, indicating that the influence of adults who quit on increased health practitioner expenditure is reduced for those living in outer regional areas. This could be because those living in rural areas generally experience poorer health outcomes due to multiple factors, such as lifestyle and access to healthcare [37].

Several interactions between education expenditure and smoking status were found. Overall, adults who quit had increased spending on education, and this was strengthened by not having children, and being male. As age increased, smokers spent more on education than ex-smokers but these differences in spending were very low. Previous research indicates that time preferences are a key component of the theory of rational addition, whereby present-oriented people are more prone to addiction and ex-smokers were less present-oriented and less impulsive than smokers [38]. Ex-smokers may be less present-oriented and more able to envisage medium- to longer-term consequences than smokers who may be more impulsive [38] and this may diminish with age. A similar explanation could be delayed reward discounting (DRD), a concept from behavioural economics that describes a specific type of impulsive decision-making reflecting how quickly a reward loses its value based on its delay in time [39]. For example, substance dependence manifests behaviourally as an individual’s preference for smaller immediate rewards at the expense of considerable benefits in the future from not using the drug. A review of DRD studies indicates strong evidence of greater DRD in individuals exhibiting addictive behaviour [39]. The lower expenditure on insurance by smokers compared to ex-smokers is consistent with a previous cross-sectional study from Australia that found similar patterns of insurance expenditure to our study between smokers and non-smokers [40]. We found that the influence of smoking on reduced insurance expenditure was strengthened by living in remote areas and being married.

Our study has several strengths. We were able to analyse a large nation-wide representative longitudinal sample of smokers and ex-smokers over a seven-year period by socioeconomic quintiles. The only previous study that has examined these groups’ expenditure longitudinally is from the US using a follow up period of 12 months [16]. Previous studies on expenditure of smokers in Australia by SEP have been based on a national cross-sectional surveys and qualitative interviews that compared them with adults who do not smoke [11, 40]. Our GEE model selection approach based on *p*-value< 0.05 is justified given that a post-hoc analysis showed that based on an average sample size of 2500 per year with compound symmetry correlation structure (corr = 0.5) across annual measure, the models can detect sufficiently small effects (i.e. an standardised effect size of 0.08, equivalent to 8% of SD) with 80% power. Our study could have been strengthened using only smokers pre- and post-cessation, however this was not possible due to insufficient numbers in the sample. We use an area-level socioeconomic measure of socioeconomic position, which may omit substantial proportions of individual variation in education and income [41]. However, there is evidence that area-based measures capture the complex relationship between various economic and social phenomena that cannot be picked up by individual-based measures [42]. Nonetheless, we have provided results by measure of income and occupation (Table 3) with very similar findings.

Because tobacco and alcohol use are highly prevalent in several other high-income countries, our findings of a reduction in alcohol expenditure associated with cessation are also of relevance outside of Australia. In the UK, for example, alcohol expenditure as a proportion of income is highest amongst the most disadvantaged [43]. Understanding how households reallocate spending when consumption of tobacco and alcohol are reduced may alleviate financial strain amongst disadvantaged groups as well as improve health. While prevalence of smoking remains high among people with mental illness [44, 45], future research could explore whether mental health impacts household expenditure of smokers and ex-smokers especially amongst disadvantaged groups.

## Conclusions

Smoking cessation not only results in direct health benefits, but also appears to have societal economic benefits. In SEIFA quintile 2, ex-smokers, had the most significant differences in spending categories. SEIFA quintiles 1 and 2 had higher spending on meals eaten out, education, motor vehicle fuel and insurance compared to smokers daily. The reduction in alcohol spending by ex-smokers overall indicates a joint health benefit that could be used to encourage policymakers, funders, primary healthcare and the alcohol and other drug sectors, to address smoking cessation more actively. Amongst low-SEP households and across the sample, spending by ex-smokers indicates positive impacts on households and increased spending in their local communities on non-tobacco products.

## Supplementary Information

**Additional file 1.**

**Additional file 2.**

## Data Availability

The survey data that support the findings of this study are available in Australian Data Archive Dataverse with the identifier 10.26193/BBOTSM

## Abbreviations

ABS: Australian Bureau of Statistics
CI: Confidence Interval
DRD: Delayed reward discounting
GEE: Generalized estimating equation
HILDA: Household, Income and Labour Dynamics in Australia
IEO: Index of Education and Occupation
IQR: Interquartile range
SEIFA: Socioeconomic Index for Areas
SEP: Socioeconomic position
