# Supplementary material for: Household expenditure of smokers and ex-smokers across socioeconomic groups: results from a large nationwide Australian longitudinal survey
Source: BMC Public Health. 2022 Sep 8;22:1706. doi: 10.1186/s12889-022-14083-y (PMC9461138; doi:10.1186/s12889-022-14083-y)
Supplement: Supplementary file 1 — Additional file 1. [file 12889_2022_14083_MOESM1_ESM.pdf]

Additional file 1

Imputation Method

The HILDA User Manual Release 18.0 states that there were three main imputation methods for the expenditure items in our study [1].

1. Carryover zeros. The population carryover method was used to determine zero and non-zero expenditure flags for non-lumpy expenditure items prior to any other imputation. (Lumpy items include cars and white goods).
2. Nearest Neighbour Regression imputation of zeros. The predicted values from a regression model were used to identify a donor from which to flag zero or non-zero imputes for the recipient. This is essentially a filter process to decide whether the case has the expense or not.
3. Nearest Neighbour Regression imputation of non-zero amounts. The predicted values from a regression model were used to identify a donor from which the reported value is taken as the imputed value for the recipient. The models and donor pools are restricted to cases with non- zero amounts. For households without any expenditure data reported in the Self-Completion Questionnaire (SCQ), a single donor for all expenditure variables collected in the SCQ was used.
4. Little and Su imputation [2]. This method incorporates (via a multiplicative model) the trend across waves (column effect), the recipient's departure from the trend (row effect), and a residual effect donated from another case with complete expenditure information for that component (residual effect). Only cases that have been enumerated in more than one wave, longitudinally linked, and have at least one wave of non-zero data available can be imputed via this method.

Imputation classes were used for some variables to ensure the donors and recipients matched on a small number of characteristics (typically equivalised household disposable income bands and the age group of the highest income earner were used). A full description of the imputation process for the expenditure variables is provided by Sun (2010) [3].

Missing expenditure data

The percentage of cases with missing expenditure data is provided in Table 1

Table 1 Households with missing expenditure data, waves 12 – 18 (%)

	Wave						
Variable	12	13	14	15	16	17	18
**Weekly household expenditure (collected in the Household Questionnaire**							
All groceries	1.4	1.3	0.9	1.0	1.3	1.2	1.0
Groceries for food and drink	1.7	1.5	1.2	1.2	1.5	1.3	1.2
Meals eaten outside the home	1.2	0.9	0.8	0.7	0.9	0.7	0.8
**Annualised household expenditure (collected in the Self completion Questionnaire**							
Groceries	17.8	17.9	17.0	18.1	15.9	15.5	14.3
Alcohol	18.8	18.6	17.6	18.8	16.5	16.4	14.7
Cigarettes and tobacco	19.3	19.3	18.1	19.2	16.7	16.7	15.0
Meals eaten out	18.2	18.4	17.4	18.5	16.1	15.8	14.3
Motor vehicle fuel	18.2	18.2	17.3	18.0	15.8	15.6	14.3
Telephone rent and calls, internet charges	18.2	18.4	17.7	18.4	16.1	15.9	14.5
Insurances	19.4	19.6	18.4	19.2	16.9	16.8	15.2
Fees paid to health practitioner	19.3	19.1	18.2	18.9	17.1	16.9	14.9
Medicines, prescriptions and pharmaceuticals	19.1	19.1	18.0	18.7	17.0	16.6	14.9
Education fees	19.2	19.6	18.5	18.9	17.2	16.9	15.1

References

1. Summerfield M, Bright S, Hahn M, La N, Macalalad N, Watson N, et al. HILDA User Manual – Release 18.: Melbourne Institute: Applied Economic and Social Research, University of Melbourne; 2019.

2. Little R, Su H. Item Non-Response in Panel Surveys. In: Kasprzyk D, Duncan GJ, Kalton G, Singh MP, editors. Panel Surveys. New York: John Wiley and Sons; 1989.
