# Supplementary material for: Household expenditure of smokers and ex-smokers across socioeconomic groups: results from a large nationwide Australian longitudinal survey
Source: BMC Public Health. 2022 Sep 8;22:1706. doi: 10.1186/s12889-022-14083-y (PMC9461138; doi:10.1186/s12889-022-14083-y)
Supplement: Supplementary file 2 — Additional file 2. [file 12889_2022_14083_MOESM2_ESM.pdf]

Additional file 2.

Table 1: Median expenditure by SEIFA quintile per annum by category ($A) averaged across waves.

		Alcohol	Meals eaten out	Groceries	Education	Clothing	Motor Vehicle Fuel	Medicine	Health Practitioner	Insurance
**Total sample**	Ex-smokers<br>Median, IQR	$1108.7<br>(0–2653.9)	$2356.4<br>(1071.7–4171.0)	$9450.0<br>(6339.5– 13231.8)	$0<br>(0–200)	$982.5<br>(404.5–1958.6)	$1850.2<br>(945.3– 3131.6)	$365.3<br>(158.3–712.3)	$571.0<br>(171.3–1348.6)	$1804.7<br>(952.9–2868.1)
	Smokers<br>Median, IQR	$1157.6<br>(0–2813.5)	$1825<br>(566.5–3214.0)	$8258.5<br>(5399.7–11732.0)	$0<br>(0–243.2)	$831.6<br>(286.6–1726.7	$1708.1<br>(840– 2954.2)	$205.0<br>(71.7–506.9)	$228.4<br>(0–674.3)	$1140<br>(265.2–2131.3)
**Quintile 1**	Ex-smokers<br>Median, IQR	$543.1<br>(0–1939.9)	$1564<br>(550.0–2826.5)	$7939.1<br>(5282.7–10799.4)	$0<br>(0–0)	$600<br>(238.22–1343.1)	$1423.4<br>(709.0– 2592.0)	$306.5<br>(112.7–527.7)	$269.7<br>(0–727.4)	$1260.0<br>(511.3–2263.3)
	Smokers<br>Median, IQR	$1087.2<br>(0–2717.5)	$1178.2<br>(0–2729.5)	$7821.0<br>(5214.0–10757.9)	$0<br>(0–85.9)	595.6<br>(196.6–1200.0)	$1280.7<br>(616.7–2466.9)	$153.7<br>(50.7–376.6)	$36.1<br>(0–404.6)	$729.3<br>(0–1600)
**Quintile 2**	Ex-smokers<br>Median, IQR	$1087.2<br>(0–2607.0)	$1963.6<br>(647.6–3302.8)	$8460.5<br>(5565.7–12131.2)	$0<br>(0–0)	$801.7<br>(335.8–1678.8)	$1728.9<br>(900.0–3000.0)	$350<br>(152.2–659.6)	$436.1<br>(40.5–1090.2)	$1597.6<br>(794.0–2662.7)
	Smokers<br>Median, IQR	$1094.3<br>(0–2717.5)	$1793.8<br>(546.5–2945.0)	$8348.6<br>(5811.6–11644.2)	$0<br>(0–154.1)	$801.7<br>(289.0–1717.9)	$1800.0<br>(945.3–2890.5)	$203.0<br>(60.0 –500.0)	$190.7<br>(0–674.3)	$1121.6<br>(243.1–2187.7)
**Quintile 3**	Ex-smokers<br>Median, IQR	$1094.3<br>(0–2607)	$2261.6<br>(1092.0–3750.3)	$8809.7<br>(5939.2–12523.4)	$0<br>(0–288.2)	$1036.0<br>(458.1–1800.0)	$2049.1<br>(1088.56–3330.4)	$354.8<br>(153.7 –635.3)	$571.0<br>(207.8–1308.2)	$1823.1<br>(1032.5–2753.4)
	Smokers<br>Median, IQR	$1386.1<br>(25.5–2813.5)	$2034.1<br>(869.0–3391.3)	$8099.5<br>(5399.7–11210.0)	$0<br>(0–328.6)	$956.45<br>(345.3–1700.7)	$1920.5<br>(1024.5–3189.9)	$211.1<br>(80.0–507.4)	$269.7<br>(0–727.4)	$1167.4<br>(480.0–2144.0)
**Quintile 4**	Ex-smokers<br>Median, IQR	$1327.5<br>(443.3–2735.2)	$2751.8<br>(1299.6–5214.0)	$10585.4<br>(7708.3–13914.4)	$0<br>(0–500)	$1151.1<br>(515.4–2302.3)	$1927.0<br>(1024.5–3545.0	$414.9<br>(203.0–829.8)	$725.0<br>(300.0–1635.2)	$2000.0<br>(1147.2–3038.5)
	Smokers<br>Median, IQR	$1662.5<br>(277.4–4246.1)	$2678.6<br>(1154.6–4418.0)	$10428.0<br>(6455.0 –14309.0)	$0<br>(0–616.3)	$1133.8<br>(458.1–2253.9)	$2364.0<br>(1280.66–3700.4)	$265.7<br>(101.5–550.0)	$500<br>(103.9–1091.1)	$1440.7<br>(486.2–2673.9)
**Quintile 5**	Ex-smokers<br>Median, IQR	$1592.155<br>(553.8–2893.5)	$2885.9<br>(1413.8–5458.9)	$10585.4<br>(7680.4–14555.4)	$0<br>(0–657.1)	$1200<br>(572.6–2748.7)	$1921.0<br>(996.4–3273.9)	$425.3<br>(202.8 –900.0)	$969.8<br>(381.6–2289.3)	$2212.5<br>(1337.8–3453.2)
	Smokers<br>Median, IQR	$2123.6<br>(543.1–3939.1)	$2751.8<br>(1178.2–5357.3)	$10565.5<br>(6607.0–15642.0)	$0<br>(0–636.1)	$1191.1<br>(453.5–2600.6)	$1840.0<br>(1055.5–3072.4)	$357.6<br>(150.0–659.6)	$571.0<br>(218.0–1283.7)	$1638.4<br>(893.3 –2673.9)

Notes: IQR; Interquartile range.
